# Ternary Ionic Liquid Analogues as Electrolytes for Ambient and Low-Temperature Rechargeable Aluminum Batteries

**DOI:** 10.1021/acsaem.4c00739

**Published:** 2024-06-20

**Authors:** Jonah Wang, Theresa Schoetz, Leo W. Gordon, Elizabeth J. Biddinger, Robert J. Messinger

**Affiliations:** Department of Chemical Engineering, The City College of New York, CUNY, New York, New York 10031, United States

**Keywords:** multivalent-ion batteries, anionic redox, electrolyte phases, liquid-state NMR spectroscopy, cyclic voltammetry, differential scanning calorimetry

## Abstract

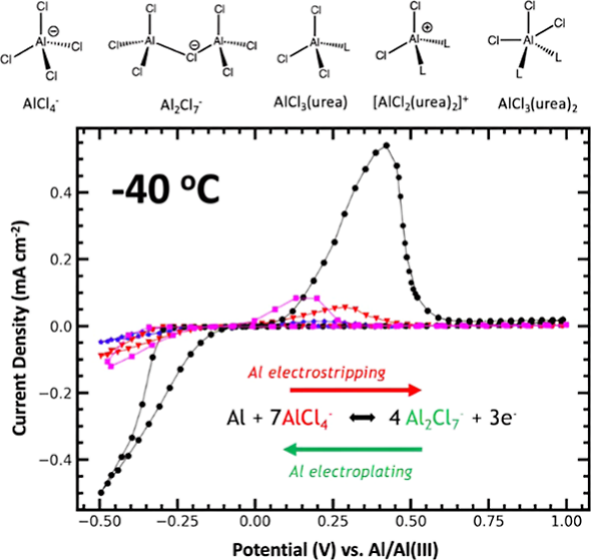

Rechargeable aluminum (Al) metal batteries are enticing for the coming generation of electrochemical energy storage systems due to the earth abundance, high energy density, inherent safety, and recyclability of Al metal. However, few electrolytes can reversibly electrodeposit Al metal, especially at low temperatures. In this study, Al electroplating and stripping were investigated from 25 °C to −40 °C in mixtures of aluminum chloride (AlCl_3_), 1-ethyl-3-methyl-imidazolium chloride ([EMIm]Cl), and urea. The ternary ionic liquid analogue (ILA) consisting of AlCl_3_–urea–[EMIm]Cl in a molar ratio of 1.3:0.25:0.75 enabled reversible Al electrodeposition at temperatures as low as −40 °C while exhibiting the highest current density and the lowest overpotential among all of the electrolyte mixtures at 25 °C, including the AlCl_3_–[EMIm]Cl binary mixture. The ILA electrolyte was further tested in a rechargeable Al–graphite battery system down to −40 °C. The addition of urea to AlCl_3_–[EMIm]Cl binary mixtures can improve the Al electrodeposition, extend the liquid temperature window, and reduce the cost.

## Introduction

Due to growing rechargeable energy storage demands, the market for affordable battery-powered vehicles, equipment, and accessories has grown substantially. Now, there is a prevailing demand for batteries that function in low-temperature environments, such as climates far from the equator, high in altitude, or in space. Current battery technology is not capable of functioning in cold environments below 0 °C and requires external thermal management for operation at lower temperatures, resulting in added system complexity and low efficiency. The reliable, widespread use of electric vehicles, for example, requires operation at −30 °C,^1^ while space applications can require operation down to −60 °C or colder.^2^

For example, an analysis of more than 10,000 electric vehicles done by Taggart^3^ showed that at only −10 °C, the energy consumption of a Li-ion battery increases up to 45% compared to room temperature; meanwhile, batteries for NASA’s CADRE (Cooperative Autonomous Distributed Robotic Exploration) project must withstand lunar temperatures, where surface temperatures can range from −232 to 120 °C and allowable flight temperatures from −20 to 75 °C.^2^ Research focused on improving the operating temperature window of Li-ion batteries often involves the addition of cosolvents and/or additives to the electrolyte. For example, cathode–electrolyte interfaces between the NMC cathode and the electrolyte containing more Li_2_CO_3_ and P–O compounds formed via addition of LiPO_2_F_2_ are shown to be compact and conductive, allowing for good cycling stability and fast Li^+^ transport, even at low temperatures.^4^ Jow et al.^5^ showed that additives affected the charge transfer at the electrodes; the LiBOB additive was found to reduce the charge transfer resistance on the cathode, while additional LiFSI salt was found to lower the activation energy of Li^+^ charge transfer at the anode compared to standard electrolytes. Smart et al.^6^ used ester cosolvents methyl propionate and ethyl propionate to improve the Li-ion battery performance at −60 °C, due to the lower molecular weights and viscosities of the added cosolvent esters. While these methods have had success in suppressing capacity loss in Li-ion battery systems, the recommended operating temperature for standard systems is still limited to ca. −20 °C;^1^ thus improvements to low-temperature performance are critical to allow for more expansive application of rechargeable batteries.

Recently, rechargeable aluminum (Al) metal batteries have been considered as an attractive alternative battery, including for low-temperature applications,^7^ due to the low cost, inherent safety, high theoretical capacities, and natural earth abundance^8^ of the aluminum anode and the graphite cathode. However, few organic solvents can plate and strip Al and those have limited applications due to their narrow electrochemical stability windows, high volatility, and low solubility of Al.^9^ Thus, the use of ionic liquids (ILs) or their derivatives, IL analogues (ILAs), has been studied as electrolytes for batteries and electrodeposition.^7−9^ ILs possess low flammability and volatility, have high thermal stability and low vapor pressure in a physical liquid state, and have tunable structural and electrochemical properties by composition alteration.^10,11^ The current gold-standard electrolyte in Al battery research is Lewis acidic mixtures of aluminum chloride (AlCl_3_), 1-ethyl-3-methyl-imidazolium ([EMIm]Cl) electrolyte. The AlCl_3_–[EMIm]Cl IL electrolyte benefits from high conductivity and a wide electrochemical stability window and is one of the most used electrolytes in Al battery research.^12^

Despite the success of the IL electrolyte, the AlCl_3_–[EMIm]Cl system has its drawbacks, including being expensive, hygroscopic, highly corrosive, and having poor low-temperature performance due to a narrow liquid temperature window.^11,13^ Lower-cost alternatives, such as Lewis acidic mixtures of AlCl_3_–urea^14^ and AlCl_3_–gamma-butyrolactone,^15^ have been explored to mitigate the cost and corrosiveness of the electrolyte, but the performance can vary greatly depending on the speciation and composition of the electrolytes.^16,17^ Additionally, any such electrolytes must dissolve the native oxide layer present on the Al metal surface to enable reversible Al electrodeposition.^9^ Overall, improving the standard Al electrolyte would drastically impact the feasibility of Al battery technology, allowing for further application of a battery composed of more sustainable materials.

Researchers have been synthesizing Al–electrolyte mixtures to enhance the electrochemical performance, including at low temperatures.^7^ Schoetz et al.^7^ investigated combinations of AlCl_3_ with different imidazolium cations and showed that AlCl_3_ (66 mol %) mixed with [EMIm]Cl/[BMIm]Cl in a molar ratio of 2:1 achieved significantly improved specific capacity retention in Al–graphite batteries down to −20 °C compared to the binary AlCl_3_–[EMIm]Cl and AlCl_3_–[BMIm]Cl counterparts. Most recently, Tsuda et al.^18^ found that varying compositions of ternary mixtures of AlCl_3_–urea–[EMIm]Cl resulted in unique Al nanoplatelet deposition, though the mechanism is still not well understood. Li et al.^19^ recently utilized AlCl_3_–urea–[EMIm]Cl ternary electrolytes in Al–graphite batteries, although at ambient temperature. Urea has garnered particular interest in electrolyte usage^11,14^ due to its vast global production volume. Urea is one of the top-produced chemicals in the world (190 million tons/year), with annual demand growing 3–4% per year,^20^ making it a practical and cost-effective alternative to [EMIm]Cl.

Thermodynamic calculations by Brunet et al.^21^ have suggested that the freezing point of an *n*-component mixture can be depressed via increasing the entropy of mixing. Zhang et al.^22^ used this method to design Li-ion electrolytes that remained in the liquid phase as low as −130 °C, while Cho et al.^23^ utilized the same calculation to develop carbonate–nitrile Li-ion electrolytes capable of maintaining the liquid form down to −110 °C. The method has also been used to predict eutectic points in benzoquinones^24^ and many other solvents.^25^ Similarly, work by Schoetz et al.^7^ on IL electrolytes for Al batteries has shown that increasing the entropy of the liquid phase has general success in depressing the freezing point. Yalkowsky^26^ summarized Carnelley’s rule and its relationship to melting point, attesting that there is a significant role for the entropy of melting, with molecules needing to have high symmetry, flexibility, and eccentricity to suppress melting. Lian and Yalkowsky^27^ tested the role high symmetry, flexibility, and eccentricity had by calculating melting points for 481 different hydrocarbons using Brunet’s method,^21^ but there has not been work published showing how well this method applies to more nonideal solutions.

Here, we show that the addition of a third component, urea, to the binary AlCl_3_–[EMIm]Cl IL significantly suppresses the freezing point beyond −80 °C and improves the performance of reversible Al electrodeposition at both low and ambient temperatures. Differential scanning calorimetry (DSC), nuclear magnetic resonance (NMR) spectroscopy, and electrochemical methods were used to characterize thermodynamic phase transitions, electrolyte speciation, and electrochemical properties of AlCl_3_–urea–[EMIm]Cl ILA electrolytes in molar ratios of 1.3:*X*:(1 – *X*) with varying compositions ranging from *X* = 0 to *X* = 1. The ternary AlCl_3_–urea–[EMIm]Cl ILA electrolyte with a molar ratio of 1.3:0.25:0.75 was determined to be the optimal composition for reversible Al metal electrodeposition down to −40 °C. Notably, this ILA electrolyte composition also exhibted the greatest current density for reversible Al electrodeposition at 25 °C. While many studies have been performed using various compositions of binary AlCl_3_–urea and AlCl_3_–[EMIm]Cl electrolytes, few have studied ternary mixtures of all three components, and none have been studied below ambient temperature. The electrochemical performance of the AlCl_3_–urea–[EMIm]Cl ILA electrolyte was also evaluated in a rechargeable Al–graphite battery system.

## Methods

### Electrolyte Preparation

AlCl_3_–urea–EMIm]Cl electrolytes were synthesized using molar ratios of 1.3:*X*:(1–*X*), where *X* is the molar ratio of urea. Electrolytes were studied with varying relative concentrations of urea and [EMIm]Cl, where *X* was 0, 0.125, 0.25, 0.50, 0.75, or 1, corresponding to urea mole percents of 0, 5.43, 10.87, 21.74, 32.61, and 43.48%, respectively. In all electrolytes, the urea + [EMIm]Cl mole percentage was always equal to 43.48%. All electrolytes were synthesized in an argon-filled glovebox (H_2_O, O_2_ < 1.0 ppm). Solutions were prepared by first mixing together carbamide (urea; 99.5%, Acros Organics) and 1-ethyl-3-methylimidazolium chloride ([EMIm]Cl; 98%, Tokyo Chemical Industry Co.) using urea/[EMIm]Cl molar ratios of 1:0, 0.125:0.875, 0.25:0.75, 1:1, 0.75:0.25, and 0:1. AlCl_3_ (Fisher Scientific, 99.999%) was then added to the mixtures such that the molar ratio of AlCl_3_/(urea + [EMIm]Cl) was 1.3:1. AlCl_3_ was slowly added to the urea–[EMIm]Cl mixture while constantly stirring. Due to the exothermic nature of the reaction, the vial was placed in a cooling device (Techne N^o^ICE Peltier cooler) that was filled with ceramic-coated cooling beads during synthesis, which were maintained at 8 °C, to mitigate thermal electrolyte decomposition. After mixing the AlCl_3_, the vial was placed on a hot plate set to 60 °C and magnetically stirred until the solution was rendered homogeneous.

### Electrochemical Measurements

Symmetric Al–Al two-electrode cells were assembled in an argon-filled glovebox (H_2_O, O_2_ < 1.0 ppm) using polytetrafluorethylene (PTFE) Swagelok unions with diameters of 6 mm. Al electrodes (0.1-mm thick, 99.99%, Alfa Aesar) were punched into 6-mm diameters and separated by a glass microfiber filter (GF/D, Whatman) and filled with 30 μL of electrolyte within the Swagelok unions. The cells were galvanostatically cycled with an Arbin LBT battery tester. Symmetric Al–Al cells were cycled in an environmental chamber (ESPEC BTZ-133) at controlled temperatures at current densities of 0.01 mA cm^–2^ with a 45 min period of applied current, alternating between positive and negative current application. The process was repeated until the range of specific temperatures had been completed, or until the cell potential exceeded ±2 V limit. Galvanostatic cycling was performed in an environmental chamber (ESPEC BTZ-133) following a schedule of 25, 0, −20, and −40 °C for 10 h each. The test resulted in approximately 6 cycles at 0.01 mA cm^–2^ for each temperature range. The maximum cell potential recorded at each temperature (excluding the first cycle, to allow for temperature equilibration) was plotted to compare the low-temperature performance between electrolyte formulations. Al–graphite cells were also constructed using PTFE Swagelok unions with diameters of 6 mm. Al anodes and graphite cathodes (80-μm thick; 90% natural graphite, Alfa Aesar, 99.9995% metal basis; 10% poly(vinylidene fluoride), Sigma-Aldrich, average molecular weight 534 000 g mol^−1^) were separated by a 7-mm diameter circle glass microfiber filter (Whatman, GF/D) soaked with 30 μL of electrolyte and cycled from 2.45 to 0.2 V at a rate of 60 mA/g.

Cyclic voltammetry (CV) measurements were performed in a three-electrode PTFE Swagelok cell to determine the reversibility, plating potential, and Coulombic efficiency (CE) of the electrolytes using a Biologic VSP-300 potentiostat. The cells contained glassy carbon (GC) as working and counter electrodes (Alfa Aesar, 0.1256 cm^2^) and an Al wire (0.5-mm tip radius, Thermo Fisher Scientific, 99.999% metal basis) as a quasi-reference electrode. Cyclic voltammograms were recorded between −0.5 and 1 V vs Al|Al(III). Three scans for each scan rate were performed to ensure reproducibility.

### Differential Scanning Calorimetry

DSC measurements were performed using a DSC Q200 (Thermal Analysis Instruments). DSC samples were prepared in an argon-filled glovebox (H_2_O, O_2_ levels <1.0 ppm). For each electrolyte sample, 8 μL was transferred to an aluminum hermetic pan, weighed, then sealed with a crimper inside the glovebox. The electrolyte samples were then transferred to the DSC Q200 and subjected to the following heat treatment. The samples went through three cycles, with one cycle consisting of being cooled initially from room temperature at a rate of 2 °C/min down to −80 °C before holding the temperature at −80 °C for 10 min. The samples were then heated to 40 °C at a rate of 5 °C/min before returning to room temperature. The samples were heated to 40 °C to erase the thermal history of the material. No significant changes in phase transition were seen between cycles, indicating the thermal history was reset between scans. The third cycle of each sample was reported. Onset melting points were determined using the heating curve, with tangent lines added via the TRIOS software (Thermal Analysis Instruments) as described in the DSC UserCom.^29^ Glass transition temperatures were determined by using the half-height method, which measures the glass transition to be the midpoint between the calculated onset and end point of the glass-transition region, where the onset and end point are calculated using tangent lines similar to the freezing point onset.^28,29^

### NMR Spectroscopy

Liquid-state NMR spectra were acquired on a Bruker AVANCE III HD 300 NMR spectrometer with a 7.05 T narrow-bore (54-mm diameter) superconducting magnet equipped with a 5-mm broadband HX Bruker probe, operating at 300.13 and 78.204 MHz for ^1^H and for ^27^Al nuclei, respectively. All liquid-state ^1^H and ^27^Al single-pulse NMR spectra were acquired using radiofrequency field strengths of 16.7 kHz (π/2 of 15 μs) and 25 kHz (π/2 of 10 μs), respectively. Liquid-state ^1^H and ^27^Al single-pulse NMR experiments were performed under quantitative conditions using recycle delays of 15 and 1 s, respectively, during which all nuclear spins relaxed to thermal equilibrium (5*T_1_, the longitudinal relaxation time). Samples were prepared in an argon-filled glovebox (H_2_O, O_2_ < 1.0 ppm) with coaxial 5 and 3-mm NMR tubes, where the inner 3-mm tube contained an isolated D_2_O locking solvent; both NMR tubes were sealed with epoxy to ensure that they were airtight.

## Results and Discussion

**Figure 1:**
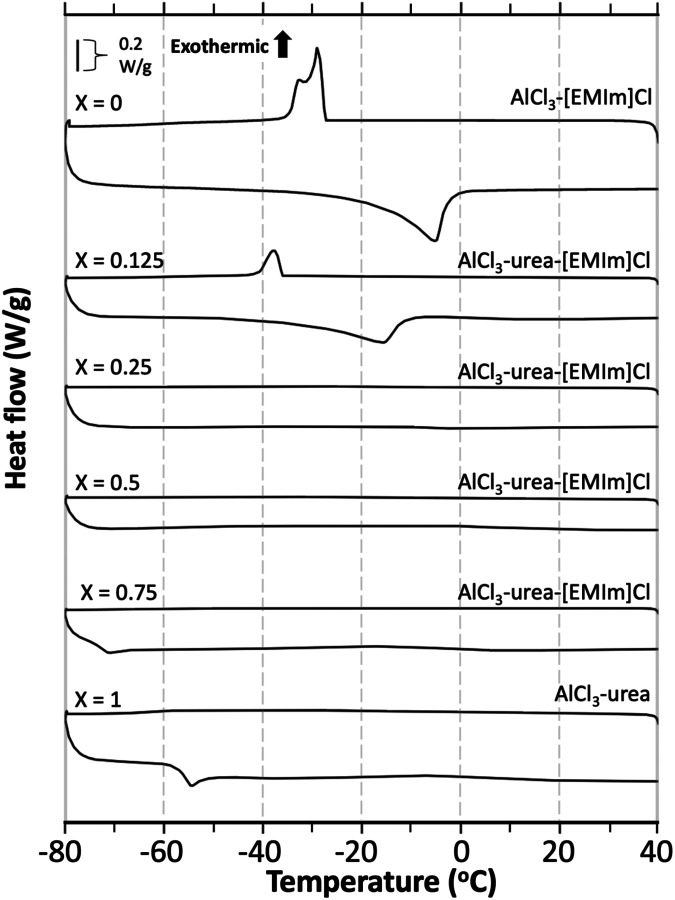
DSC thermograms of AlCl_3_–urea–[EMIm]Cl electrolytes with molar ratios of 1.3:*X*:(1-*X*), where *X* = 0, 0.125, 0.25, 0.5, 0.75, and 1.0.

DSC experiments were conducted to investigate the hypothesis that the ternary mixtures of AlCl_3_, [EMIm]Cl, and urea could maintain their liquid form down to lower temperatures than either of the binary AlCl_3_ mixtures (Figure 1). In Figure 1, the binary AlCl_3_–[EMIm]Cl at a 1.3:1 ratio exhibits a melting point at −17 °C, determined as described in the Methods section. Ferrara et al.^13^ reported a temperature of −20.15 °C for the AlCl_3_–[EMIm]Cl at a 1.3:1 ratio. This slight difference in the phase change onset may be due to impurities. Note that multiple cycles were performed in this work to prevent supercooling.^30^

In the ternary mixtures, the electrolytes with urea contents of *X* = 0.25 and 0.5 appear to have no visible phase transitions, while the electrolyte with *X* = 0.125 has a melting point of −33 °C. The electrolyte with *X* = 0.75 exhibits a glass-transition temperature at −72 °C, calculated by using the half-height method as described above. DSC thermograms with tangent and half-height lines for determining the freezing and glass transition temperatures can be found in Figure S1. The binary AlCl_3_–urea (1.3:1) (*X* = 0) electrolyte shows a much higher glass transition at −56 °C. No low-temperature DSC data is available in the literature for AlCl_3_–urea (1.3:1).

Based solely on the DSC data, the electrolyte mixtures with urea contents of *X* = 0.25 and 0.5 appear to be the most promising, as maintaining a liquid form at lower temperatures favors enhanced ion transport properties. It is believed that an excess of [EMIm]^+^ in mixtures with less urea resulted in higher concentrations of a largely asymmetric molecule.^7^ In the thermogram of the electrolyte with *X* = 0.125, the slight addition of urea results in a melting point that is 16 °C lower than that of binary AlCl_3_–[EMIm]Cl (1.3:1) (*X* = 0). The electrolyte with *X* = 0.75 also exhibits a 16 °C lower glass-transition temperature than the binary AlCl_3_–urea (1.3:1) (*X* = 1) electrolyte. The lack of molecular symmetry along with increased entropy from the additional species in the IL appears to inhibit the necessary ordering of molecules that occurs during the freezing or glass-transition process. The DSC thermograms support the idea that adding bulky cations ([EMIm]^+^) along with organic molecules (urea) in a ternary mixture would result in a depressed phase transition when subjected to subzero temperatures.

The depressed melting point of the electrolyte AlCl_3_–urea–[EMIm]Cl (1.3:0.25:0.75, *X* = 0.25) is interesting considering the work by Cerajewski et al.,^31^ who measured that the binary urea–[EMIm]Cl mixture (i.e., with no AlCl_3_) had a minimum melting point when the molar ratio of urea/[EMIm]Cl was 25:75. This molar ratio was identical to that in the ternary electrolyte with *X* = 0.25. Interestingly, molecular dynamics simulations showed that the [EMIm]^+^ molecules in binary mixtures of urea/[EMIm]Cl with 72.5:27.5 molar ratios have lower mean-squared- displacements compared to the urea molecules, while the [EMIm]^+^ molecules in urea/[EMIm]Cl with 25:75 molar ratios have higher relative displacements, indicating a superior mobility of [EMIm]^+^ cations when the molar ratio of urea/[EMIm]Cl is 25:75.^31^

**Figure 2:**
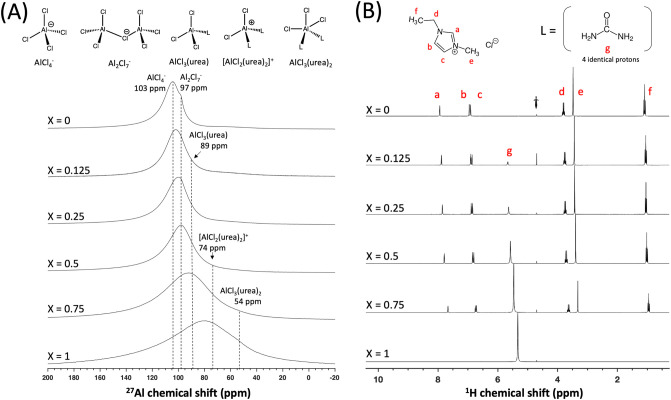
Liquid-state (A) ^27^Al and (B) ^1^H single-pulse NMR spectra of AlCl_3_–urea–[EMIm]Cl electrolytes with molar ratios of 1.3:*X*:(1-*X*), where *X* = 0, 0.125, 0.25, 0.5, 0.75, and 1.0. Molecular structures of aluminum- and proton-containing species are shown above (A) and (B), respectively, where *L* represents urea.

To better understand the different species present in the electrolyte mixtures and their local environments, liquid-state ^27^Al and ^1^H single-pulse NMR experiments were acquired under quantitative conditions (Figure 2). The ^27^Al and ^1^H single-pulse NMR spectra of the binary mixtures (*X* = 0 and 1) are both in good agreement with the literature.^13,14,16^ The ^27^Al single-pulse spectrum of the AlCl_3_–[EMIm]Cl (1.3:1, *X* = 0) electrolyte shows two ^27^Al signals at 103.7 and 97.7 ppm, which are due to AlCl_4_^–^ and Al_2_Cl_7_^–^, respectively.^13^ The addition of urea to the binary AlCl_3_–[EMIm]Cl mixture results in the generation of additional neutral and cationic species, specifically AlCl_3_(urea), [AlCl_2_(urea)_2_]^+^, and AlCl_3_(urea)_2_ complexes, which have ^27^Al shifts of 89, 74, and 54 ppm, respectively.^14,17^ As discussed below, rapid chemical exchange between these species results in significant ^27^Al broadening. As the urea content increases, the ^27^Al chemical shifts move to lower frequencies, indicating larger concentrations of the AlCl–urea complexes, as expected. In the ^1^H single-pulse spectra, the urea ^1^H signal shifts to lower frequencies as the urea content increases; this shift reflects the higher average content of the cationic [AlCl_2_(urea)_2_]^+^ species, which is in agreement with the ^27^Al NMR spectra. The molar ratios of urea/[EMIm]Cl in the different electrolyte mixtures are quantitatively consistent with their expected relative ^1^H integrated signal intensities.

In the ^27^Al single-pulse NMR spectra, the ^27^Al signal broadening with increasing urea content indicates that the complexed Al–urea species undergo rapid chemical exchange compared with the differences in their NMR frequencies. This rapid exchange between species reduces the spectral resolution. Notably, the shift of the ^27^Al signals to lower frequencies indicate fewer Al_2_Cl_7_^–^ species are present when the urea/[EMIm]Cl ratio increases, which is a crucial species for the electrodeposition of aluminum metal.^9−11^^27^Al NMR spectra acquired by Malik et al.^32^ at −10 °C in AlCl_3_–urea binary mixtures showed significant changes in aluminum speciation with changing AlCl_3_/urea molar ratios. The variation in electrolyte speciation could result in a different and/or multiple electrodeposition pathway(s), as Al-urea complexes such as [AlCl_2_(urea)_2_]^+^ have also been proposed to enable Al electrodeposition in addition to chloroaluminate Al_2_Cl_7_^–^ anions.^13,14^ Gordon et al.^16^ used thermochemical calculations to show that while these Al-complexed species can participate in Al electrodeposition reactions in AlCl_3_–urea mixtures, electrodeposition of Al metal by the Al_2_Cl_7_^–^ species^16,33^ is more favorable based on Gibbs free energy. Increasing the urea concentration resulted in a clear increase in the broad peak at 74 ppm, attributed to the [AlCl_2_(urea)_2_]^+^ complex. Researchers have proposed an alternate Al electrodeposition pathway,^14^ via [AlCl_2_(urea)_2_]^+^, but recent work done on electrolytes with only [AlCl_2_(urea)_2_]^+^ and no Al_2_Cl_7_^–^ have not shown any capability of reversible galvanostatic cycling against an Al metal anode.^16^

**Figure 3:**
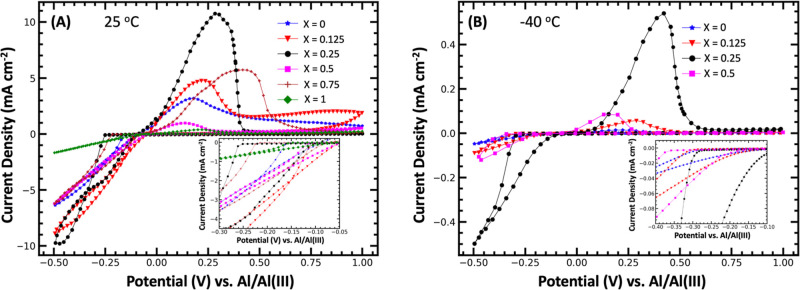
CV performed at 10 mV/s and (A) 25 or (B) −40 °C using AlCl_3_/urea/[EMIm]Cl electrolytes with molar ratios of 1.3:*X*:(1-*X*), where *X* = 0. 0.125, 0.25, 0.5, 0.75, and 1.0 or *X* = 0, 0.125, 0.25, and 0.5, respectively. A three-electrode cell was used with GC working and counter electrodes and an Al wire quasi-reference electrode. The electrolyte mixture with *X* = 0.25 exhibits the greatest current densities for Al electroplating and electrostripping at both 25 and −40 °C.

CV was performed in three-electrode cells at 25 °C on all electrolytes to confirm that they enable reversible Al electrodeposition on GC substrates (Figure 3a; stacked plots are shown in Figure S2). Redox peaks corresponding to Al electroplating and electrostripping were observed in all of the electrolytes. Onset plating potentials are defined as the potential at which the current density exceeded −0.1 mA cm^–2^ at 25 °C and −0.05 mA cm^–2^ at −40 °C. In the binary AlCl_3_–[EMIm]Cl (1.3:1, *X* = 0) electrolyte, the plating peak had an onset potential of −0.16 V. When urea was added (*X* = 0.125), the onset potential was −0.11 V. When the urea content was increased to the electrolyte with *X* = 0.25, the onset potential extended to −0.26 V. In the equimolar solution of urea–[EMIm]Cl (*X* = 0.5), the onset potential receded to −0.12 V. For the electrolyte with *X* = 0.75, the onset potential was −0.22 V, while in the binary AlCl_3_–urea (1.3:1, *X* = 1) electrolyte the onset potential was equal to the *X* = 0 electrolyte at −0.16 V. The nucleation loops for the Al plating associated with the reduction current have not only had varied onset potentials but also line shapes, suggesting a different growth model during the electrodeposition process.

Cyclic voltammograms were also integrated with respect to time to quantify the charge transfer and CE. The binary AlCl_3_–[EMIm]Cl (1.3:1, *X* = 0) electrolyte has a modest quantity of charge transferred during electroplating (73.16 mA s), associated with the area of the electroplating peak (averaged over three scans), while the corresponding stripping peak was smaller (42.84 mA s), resulting in a CE of 59%.

With the addition of urea, the electrolyte with *X* = 0.125 resulted in a significant increase in the electroplating charge, up to 114.5 mA s, but still a poor CE (55%), with a corresponding electrostripping charge of 62.51 mA s. Interestingly, increasing the urea concentration to *X* = 0.25 dramatically improved the amount of charge associated with electroplating (156.6 mA s) and stripping (119.58 mA s). When urea was concentrated beyond *X* = 0.25, the charge transfer associated with Al electrodeposition decreased significantly. The electrolyte with *X* = 0.5 had a plating charge of 74.70 mA s but a corresponding stripping charge of only 14.26 mA s, yielding the lowest CE (19%) of all electrolytes tested. Surprisingly, while the electrolyte with *X* = 0.75 resulted in less charge during the electroplating process (68.88 mA s), it was very efficient, recovering 63.17 mA s during the corresponding stripping process (92% CE). The electrolyte with *X* = 0.75 had the highest CE of all of the electrolytes tested. The binary AlCl_3_–urea (1.3:1, *X* = 1) electrolyte resulted in a significant drop in charge during electrodeposition. This electrolyte had the lowest plating charge transferred (19.56 mA s) and stripping charge transferred (6.02 mA s) of all of the electrolytes tested. A summary of the charge transferred for Al electrodeposition and stripping at 25 °C, as well as the resulting CEs, is given in Table S1.

With increasing urea content, the electroplating charge is a maximum in the electrolyte containing urea mole fractions of *X* = 0.25 (156.6 mA s), with the electrolyte containing *X* = 0.125, displaying the second-most electroplating charge transfer (114.5 mA s). Overall, the cyclic voltammograms show that adding small quantities of urea (*X* = 0.125 and 0.25) can improve the yield of Al electrodeposition. While these electrolytes have fewer AlCl_4_^–^ and Al_2_Cl_7_^–^ chloroaluminate anions, which are more favorable for Al electrodeposition compared to Al–urea complexes, the addition of urea thus enhances the liquid-temperature window and appears to improve ion transport properties. The observation of superior charge transfer in the electrolyte with *X* = 0.25 is partly corroborated by the lack of observable liquid–solid thermal transitions observed with DSC (Figure 1). Other variables, such as the onset potential and CE, do not follow monotonic trends with increasing urea content.

The difference in onset potentials could be attributed to differences in the electroplating mechanisms. Abbott et al.^34^ found that the specific concentrations of AlCl_4_^–^ and Al_2_Cl_7_^–^ in solution resulted in different growth models of Al metal and thus different onset potentials for Al electrodeposition. The electrostripping sweep of the electrolytes showed different shapes, indicating that multiple different stripping processes may be occurring. These different CV shapes could be the result of different morphologies and surface energies of the deposited Al metal.^10,35^ Interestingly, the work by Tsuda et al.^18^ utilizing ternary AlCl_3_–urea–[EMIm]Cl mixtures in 1.5:*X*:(1 – *X*) molar ratios showed that the binary AlCl_3_–[EMIm]Cl mixture resulted in the greatest current for the Al electrodeposition process at 60 °C, followed by the electrolyte with a molar ratio of urea/[EMIm]Cl of 25:75. Ferrara et al.^13^ and Paterno et al.^17^ have shown that speciation and conductivity in AlCl_3_–amide and AlCl_3_–[EMIm]Cl electrolytes can vary dramatically based on different factors, including the temperature and AlCl_3_ concentration in the electrolyte. Schoetz et al.^35^ also showed that these small changes in AlCl_3_ concentration result in cyclic voltammograms with significantly different peak potentials, CEs, and different surface-level Al depositions. The CE and line shape reported by Schoetz et al.^35^ for AlCl_3_–[EMIm]Cl (1.22:1) (63% CE) are in close agreement with those of the AlCl_3_–[EMIm]Cl (1.3:1) (*X* = 0) electrolyte (59% CE) reported in this study.

Cyclic voltammograms were also performed at −40 °C (Figure 3b; stacked plots are shown in Figure S3). Only the lower urea concentration electrolytes were capable of any observable Al electrodeposition (Figure 3b), while the overall redox peaks were much smaller in intensity. At −40 °C, the binary AlCl_3_–[EMIm]Cl (1.3:1) (*X* = 0) electrolyte showed little to no Al deposition, with only a minor electroplating charge transfer (0.47 mA s) and similarly sparse electrostripping (0.16 mA s) peaks. In contrast, the electrolyte with *X* = 0.125 appeared to be a slight improvement over the AlCl_3_–[EMIm]Cl electrolyte, although there was still very little observable electroplating (0.82 mA s) or stripping (0.42 mA s). Both electrolytes with *X* = 0 and 0.125 had a slight decrease in CE as well. Increasing the ratio of urea/[EMIm]Cl to *X* = 0.25 resulted in a dramatic increase in Al electroplating (4.3 mA s) and stripping (3.8 mA s) capability on GC at −40 °C, as well as a surprising increase in CE (89%) compared to the same electrolyte at 25 °C (77%) (Figure 3b). However, increasing the urea content beyond *X* = 0.25 resulted in less electrodeposition. The electrolyte with *X* = 0.5 saw poor electroplating (1.17 mA s) and stripping (0.72 mA s) charge transferred. Additional urea beyond the electrolyte with *X* = 0.5 resulted in no redox activity at −40 °C. A summary of the charge transferred and CEs at −40 °C can be found in Table S2.

Overall, the data suggests that to electrodeposit Al at low temperatures, not only the chloroaluminate electrolytes must be phase-stable and have sufficient ion transport properties but they also must contain sufficient concentrations of electroactive chloroaluminate ions, namely, AlCl_4_^–^ and Al_2_Cl_7_^–^. The binary AlCl_3_–[EMIm]Cl (1.3:1, *X* = 0) electrolyte had sufficient AlCl_4_^–^ and Al_2_Cl_7_^–^ ions but lacked liquid-phase stability at lower temperatures (Figure 1). Too high of a concentration of urea resulted in a more phase-stable electrolyte (Figure 1) (*X* = 0.5 and 0.75) but lacked sufficient concentrations of AlCl_4_^–^ and Al_2_Cl_7_^–^ ions. This observation is corroborated by the liquid-state ^27^Al single-pulse NMR spectra, which show an increase in Al–urea-complexed species and a decrease in AlCl_4_^–^ and Al_2_Cl_7_^–^ as the urea concentration was increased (Figure 2a).

### Al Electrodeposition at Low Temperatures

**Figure 4:**
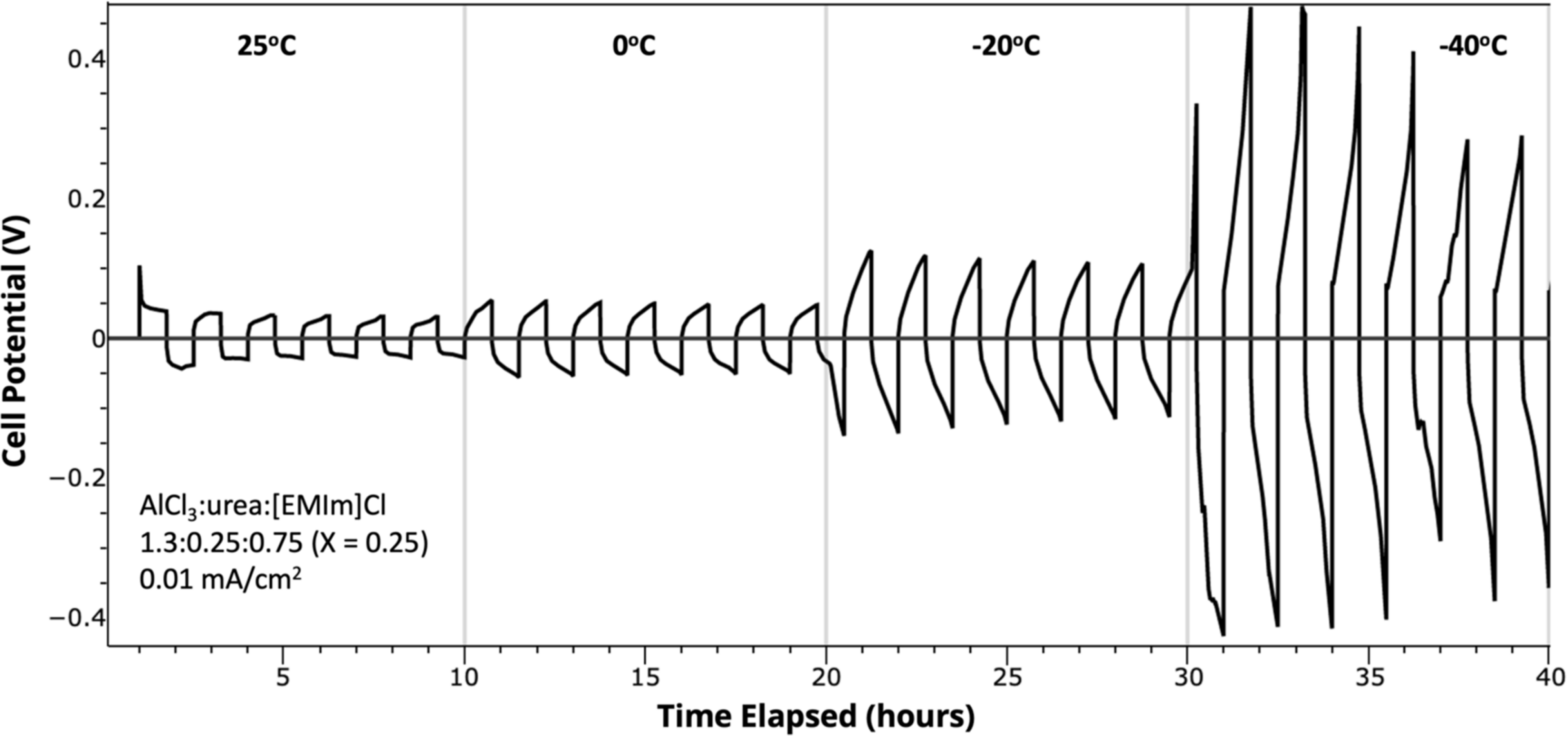
Variable-temperature galvanostatic electroplating and stripping of an Al–Al symmetric cell using an AlCl_3_–urea–[EMIm]Cl electrolyte with a molar ratio of 1.3:0.25:0.75 (*X* = 0.25) and a current density of 0.01 mA cm^–2^.

Variable-temperature galvanostatic Al electroplating and stripping experiments were performed in the AlCl_3_–urea–[EMIm]Cl electrolyte with *X* = 0.25 (Figure 4) in an Al–Al symmetric cell. The cell potential was characterized by the overpotential, which is defined as the extent of departure from the thermodynamic equilibrium potential (here, 0 V vs Al/Al(III)) when current was passed through the cell.^36^ The data shows an increase in overpotential as the temperature decreases, although even at −40 °C the electrolyte enables reversible Al electrodeposition. Within each temperature regime, the overpotential decreases slightly as the cycles progress, particularly at −40 °C.

Additionally, the overpotential for the first cycle was always the highest regardless of the temperature range. This observation could be a result of the electrolyte having different deposition morphologies, depending on the solvation structure in the first cycle. As mentioned earlier, Tsuda et al.^18^ recently showed that certain compositions of ternary mixtures of AlCl_3_–urea–[EMIm]Cl resulted in unique deposition morphologies (Al nanoplatelets) on the surface of the Al electrode, and these morphologies allow for more favorable conditions during the electrodeposition process, although they did not explore this effect at ambient or subambient temperatures. Malik et al.^32^ showed differences in speciation via NMR deconvolutions of different Lewis acidity AlCl_3_–urea mixtures, while Paterno et al.^17^ explored both the concentration and temperature, noting differences in speciation when either was varied.

### Low-Temperature Overpotentials

**Figure 5:**
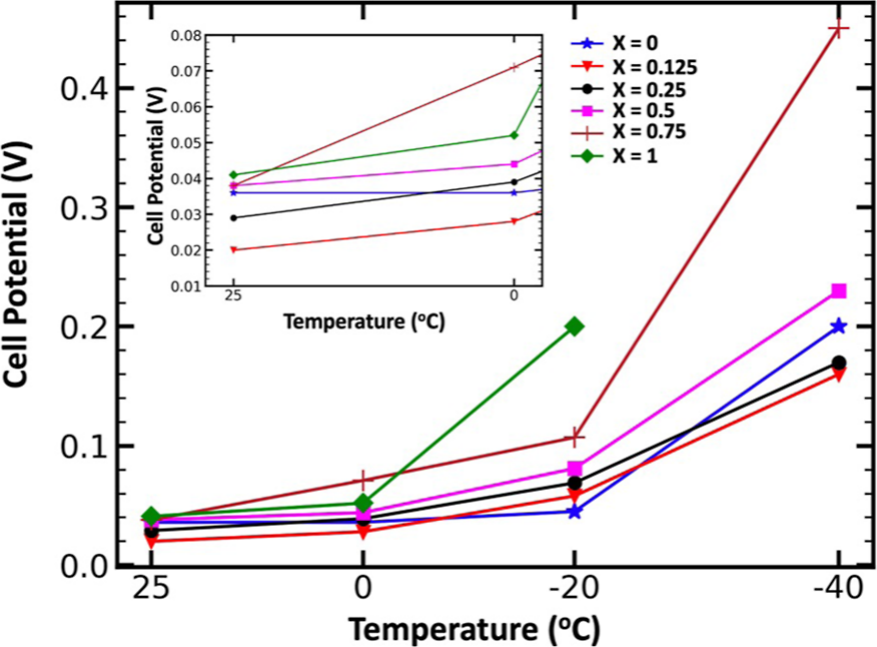
Maximum recorded overpotentials of AlCl_3_–urea–[EMIm]Cl electrolytes with molar ratios of 1.3:*X*:(1-*X*), where *X* = 0, 0.125, 0.25, 0.5, 0.75, and 1.0, in Al–Al symmetric cells at temperatures of 25, 0, 20, and −40 °C and a current density of 0.01 mA cm^–2^. Lower overpotentials were measured for the ternary AlCl_3_–urea–[EMIm]Cl electrolyte compositions with *X* = 0.125 and 0.25 at −40 °C compared to the binary AlCl_3_–[EMIm]Cl (1.3:1, *X* = 0) electrolyte.

Variable-temperature galvanostatic Al electroplating and stripping experiments were performed on all electrolytes, and the maximum overpotentials were recorded at each temperature (Figure 5). For the electrolyte with *X* = 1 at −40 °C, the cell potential reached a 2 V overpotential limit, the experiment was halted, and no data was recorded. In a comparison of electrolyte formulations, at 25 °C and 0.01 mA cm^–2^, the binary AlCl_3_–urea (1.3:1) (*X* = 1) electrolyte has the highest overpotential of 41 mV, while the binary AlCl_3_–[EMIm]Cl (1.3:1) (*X* = 0) electrolyte had an overpotential of 36 mV, and the electrolyte with *X* = 0.25 reached 0.025 V. Below −20 °C, the AlCl_3_–urea (1.3:1) electrolyte did not exhibit any electrodeposition capabilities, while all ternary compositions were able to reversibly electrodeposit Al down to −40 °C. Upon increase of the urea content in the AlCl_3_–urea–[EMIm]Cl electrolytes, the overpotential at −40 °C decreased until the electrolyte with *X* = 0.25, after which further addition of urea caused the overpotential to increase to greater than that of the AlCl_3_–[EMIm]Cl (1.3:1) (*X* = 0) electrolyte. The greatest overpotential was 410 mV in the electrolyte with *X* = 0.75 compared to the lowest overpotential of 160 mV in the electrolyte with *X* = 0.125. Both the electrolyte with *X* = 0.25 and 0.125 exhibited lower overpotentials than the AlCl_3_–[EMIm]Cl (1.3:1) (*X* = 0) electrolyte at −40 °C and were even capable of plating and stripping down to temperatures as cold as −70 °C in variable-rate galvanostatic plating and stripping tests (Figure S4), something none of the other mixtures were capable of doing. Interestingly, the overpotential at −40 °C for the electrolyte with *X* = 0.125 was only 10 mV less than that of the electrolyte with *X* = 0.25.

The high overpotentials of binary AlCl_3_–urea (1.3:1, *X* = 1) agree well with the cyclic voltammogram results at 25 °C, in which the electrolyte with *X* = 0 had the lowest charge transferred. However, the electrolyte with *X* = 0.25, which performed the best on the GC substrate, had a greater overpotential (29 mV) than the electrolyte with *X* = 0.125 (20 mV) at 25 °C in the Al–Al symmetric cells. Additionally, both the electrolyte with *X* = 0.125 and 0.25 had lower overpotentials than the binary AlCl_3_–[EMIm]Cl (1.3:1, *X* = 0) electrolyte (36 mV) at 25 °C on the Al substrate and transferred more charge than the electrolyte with *X* = 0 both at 25 °C and −40 °C. At −40 °C, the electrolytes with urea content beyond *X* = 0.5 did not exhibit observable redox data on a GC substrate, while all ternary mixtures were capable of electroplating and stripping in the Al–Al symmetric system down to −40 °C. Additionally, the overpotential of the electrolyte with *X* = 0.125 was lower than that of the electrolyte with *X* = 0.25 at every temperature point in the Al–Al symmetric system but exhibited significantly less current density than the electrolyte with *X* = 0.25 on the GC substrate, both at 25 °C (114.5 vs 156.6 mA s) and −40 °C (0.82 vs 4.3 mA s). The differences in the cyclic voltammogram and overpotential results suggest the presence of the Al electrode makes reversible Al electrodeposition more facile, even though the reaction should only need Al_2_Cl_7_^–^ to occur. Overall, the electrolytes with *X* = 0.125 and 0.25 enabled improved Al electrodeposition of Al at −40 and 25 °C, compared to the binary AlCl_3_–[EMIm]Cl (1.3:1, *X* = 0). The overpotential data in Figure 5 corroborates with the low-temperature cyclic voltammograms in Figure 3b. The lower concentrations of urea allow for lower overpotentials at −40 °C, and increasing the concentration increased the overpotential at −40 °C, except for the binary AlCl_3_–urea (1.3:1, *X* = 1), which could not plate and strip at −40 °C. Comparing the overpotential data in Figure 5 to the NMR data in Figure 2a shows that the electrodeposition with higher concentrations of Al–urea-complexed species is less favorable at low temperatures. There is increased complexation observed in the liquid-state ^27^Al single-pulse NMR spectra as the urea concentration increases, resulting in less redox current.

### Cycling in Al–Graphite Batteries

**Figure 6:**
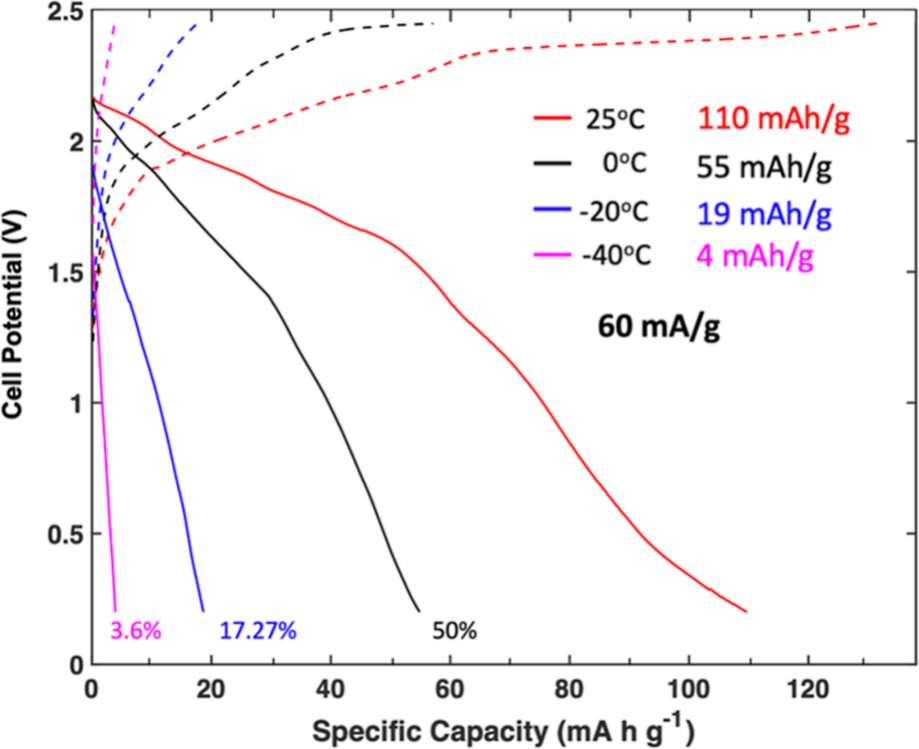
Galvanostatic cycling of Al–graphite cells using a ternary AlCl_3_–urea–[EMIm]Cl ILA electrolyte with a molar ratio of 1.3:0.25:0.75 (*X* = 0.25) at 60 mA g^–1^ and temperatures ranging from 25 to −40 °C.

To further investigate the application of the ternary AlCl_3_–urea–[EMIm]Cl ILA electrolyte with *X* = 0.25, we galvanostatically cycled Al–graphite cells at a current density of 60 mA g^–1^ at varying temperatures (Figure 6) to study how the Al electrodeposition performance of the electrolyte translated to a rechargeable full cell system. At 25 °C, the electrolyte with *X* = 0.25 retained modest specific capacity with approximately 110 mA h g^–1^ achieved at 60 mA g^–1^. Upon cooling to 0 °C, the specific capacity was reduced by ∼50%, with the battery achieving only 55 mA h g^–1^. The specific capacity further dropped to ∼17% of the room-temperature capacity at −20 °C, with only 19 mA h g^–1^, and at −40 °C it is only 4 mA h g^–1^. In comparison, Schoetz et al.^7^ showed capacity retention in Al–graphite cells at using ternary mixtures to be superior to binary ones as well, being able to retain at best 87% of capacity down to −20 °C, but similarly showed a massive drop in performance from −20 °C (87%) to −40 °C (26%) at 10 mA g^–1^, even in the best-performing electrolyte.

From comparing full-cell data to the Al–Al symmetric cells, the capacity reduction is far more dramatic in an Al–graphite full cell compared to the overpotential increase in the Al–Al symmetric cells. Despite the electrolyte with *X* = 0.25 being more thermally stable (Figure 1) and having the appropriate chloroaluminate electroactive species (Figure 2a), there appears to be other reasons for the decrease in specific capacity retention at lower temperatures beyond liquid-phase stability and favorable Al electrodeposition. The electrolyte may not be limited by just ion transport and Al electrodeposition but also by how well it can intercalate AlCl_4_^–^ into the graphite cathode at low temperatures. Schoetz et al.^7^ in low-temperature Al batteries also hypothesized that the drop is due to interactions between cations and the graphite surface.

## Conclusions

We demonstrate for the first time the benefit of adding urea to a Lewis acidic AlCl_3_–[EMIm]Cl electrolyte with regards to its ability to reversibly electrodeposit Al metal at both low and ambient temperatures. Lewis acidic AlCl_3_–urea–[EMIm]Cl ILAs were studied using molar ratios of 1.3:*X*:(1-*X*) with varying compositions from *X* = 0 to *X* = 1. DSC measurements of the AlCl_3_–urea–[EMIm]Cl electrolytes showed that the electrolytes with *X* = 0.25 and 0.5 did not reveal visible phase transitions down to −80 °C, establishing that the addition of a third species can effectively suppress phase transitions in binary chloroaluminate IL electrolytes. Liquid-state ^27^Al and ^1^H NMR spectra revealed electrolyte speciation as a function of the urea content. All electrolytes were capable of reversible Al electrodeposition on a GC substrate at room temperature, while the ternary AlCl_3_–urea–[EMIm]Cl ILA electrolyte with *X* = 0.25 transferred greater charge for both Al electroplating and stripping compared with the binary AlCl_3_–[EMIm]Cl electrolyte. At −40 °C, the electrolyte with *X* = 0.25 exhibited significantly greater charge transfer for reversible Al electrodeposition than all other electrolytes while also showing among the lowest overpotentials for galvanostatic Al electrodeposition on an Al substrate. This work demonstrates that adding urea to AlCl_3_–[EMIm]Cl binary mixtures can improve their ability to reversibly electrodeposit Al metal at low and ambient temperatures while reducing the cost.

## Supporting Information Available

DSC thermograms with calculations, CV curves of all samples, quantitative analyses of CV measurements, and variable-temperature galvanostatic reversible Al electrodeposition down to −70 °C (PDF)

## References

[ref1] Gupta A.; Manthiram A. Designing advanced lithium-based batteries for low-temperature Conditions. Adv. Energy Mater. 2020, 10 (38), 2001972 10.1002/aenm.202001972. 34158810 PMC8216142

[ref2] Jones J. P.; Smart M. C.; Krause F. C.; West W. C.; Brandon E. J. Batteries for robotic spacecraft. Joule 2022, 6 (5), 923–928. 10.1016/j.joule.2022.04.004.

[ref3] Taggart J. Ambient Temperature Impacts on Real-World Electric Vehicle Efficiency & Range. In Proceedings of the IEEE Transportation and Electrification Conference and Expo; IEEE: Chicago, IL, USA, 2017; pp 22–24.

[ref4] Piao N.; Gao X.; Yang H.; Guo Z.; Hu G.; Cheng H. M.; Li F. Challenges and development of lithium-ion batteries for low temperature environments. Etransportation 2022, 11, 100145 10.1016/j.etran.2021.100145.

[ref5] Jow T. R.; Delp S. A.; Allen J. L.; Jones J. P.; Smart M. C. Factors limiting Li+ charge transfer kinetics in Li-ion batteries. J. Electrochem. Soc. 2018, 165 (2), A361–A367. 10.1149/2.1221802jes.

[ref6] Smart M. C.; Ratnakumar B. V.; Chin K. B.; Whitcanack L. D. Lithium-ion electrolytes containing ester cosolvents for improved low temperature performance. J. Electrochem. Soc. 2010, 157 (12), A1361 10.1149/1.3501236.

[ref7] Schoetz T.; Xu J. H.; Messinger R. J. Ionic Liquid Electrolytes with Mixed Organic Cations for Low-Temperature Rechargeable Aluminum–Graphite Batteries. ACS Appl. Energy Mater. 2023, 6 (5), 2845–2854. 10.1021/acsaem.2c03762.

[ref8] Zhang Y.; Liu S.; Ji Y.; Ma J.; Yu H. Emerging non aqueous aluminum-ion batteries: challenges, status, and perspectives. Adv. Mater. 2018, 30 (38), 1706310 10.1002/adma.201706310. 29920792

[ref9] Tsuda T.; Stafford G. R.; Hussey C. L. Review—Electrochemical Surface Finishing and Energy Storage Technology with Room-Temperature Haloaluminate Ionic Liquids and Mixtures. J. Electrochem. Soc. 2017, 164 (8), H5007–H5017. 10.1149/2.0021708jes.

[ref10] Liu B.; Jin N. The applications of ionic liquid as functional material: a review. Curr. Org. Chem. 2016, 20 (20), 2109–2116. 10.2174/1385272820666160527101844.

[ref11] Zhang M.; Kamavarum V.; Reddy R. G. New electrolytes for aluminum production: Ionic liquids. Jom 2003, 55 (11), 54–57. 10.1007/s11837-003-0211-y.

[ref12] Zhu N.; Zhang K.; Wu F.; Bai Y.; Wu C. Ionic liquid-based electrolytes for aluminum/magnesium/sodium-ion batteries. Energy Mater. Adv. 2021, 2021, 9204217 10.34133/2021/9204217.

[ref13] Ferrara C.; Dall’Asta V.; Berbenni V.; Quartarone E.; Mustarelli P. Physicochemical characterization of AlCl_3_–1-Ethyl-3-methylimidazolium chloride ionic liquid electrolytes for aluminum rechargeable batteries. J. Phys. Chem. C 2017, 121 (48), 26607–26614. 10.1021/acs.jpcc.7b07562.

[ref14] Angell M.; Zhu G.; Lin M. C.; Rong Y.; Dai H. Ionic liquid analogs of AlCl_3_ with urea derivatives as electrolytes for aluminum batteries. Adv. Funct. Mater. 2020, 30 (4), 1901928 10.1002/adfm.201901928.

[ref15] Wen X.; Liu Y.; Xu D.; Zhao Y.; Lake R. K.; Guo J. Room-temperature electrodeposition of aluminum via manipulating coordination structure in AlCl_3_ solutions. J. Phys. Chem. Lett. 2020, 11 (4), 1589–1593. 10.1021/acs.jpclett.0c00256. 32037830

[ref16] Gordon L. W.; Wang J.; Messinger R. J. Revealing impacts of electrolyte speciation on ionic charge storage in aluminum-quinone batteries by NMR spectroscopy. J. Magn. Reson. 2023, 348, 107374 10.1016/j.jmr.2023.107374. 36706465

[ref17] Paterno D.; Rock E.; Forbes A.; Iqbal R.; Mohammad N.; Suarez S. Aluminum ions speciation and transport in acidic deep eutectic AlCl_3_ amide electrolytes. J. Mol. Liq. 2020, 319, 114118 10.1016/j.molliq.2020.114118.

[ref18] Tsuda T.; Miyakawa R.; Kuwabata S. Aluminum Nanoplatelet Electrodeposition in AlCl_3_–1-Ethyl-3- Methylimidazolium Chloride–Urea Melts. J. Electrochem. Soc. 2022, 169 (9), 092520 10.1149/1945-7111/ac91fc.

[ref19] Li J.; Tu J.; Jiao H.; Wang C.; Jiao S. Ternary AlCl_3_-urea-[EMIm] Cl ionic liquid electrolyte for rechargeable aluminum- ion batteries. J. Electrochem. Soc. 2017, 164 (13), A3093–A3100. 10.1149/2.0811713jes.

[ref20] Antonetti E.; Iaquaniello G.; Salladini A.; Spadaccini L.; Perathoner S.; Centi G. Waste-to-chemicals for a circular economy: the case of urea production (waste- to-urea). ChemSusChem 2017, 10 (5), 912–920. 10.1002/cssc.201601555. 27958665

[ref21] Brunet L.; Caillard J.; André P. Thermodynamic calculation of n-component eutectic mixtures. Int. J. Mod. Phys. C 2004, 15 (05), 675–687. 10.1142/S0129183104006121.

[ref22] Zhang W.; Xia H.; Zhu Z.; Lv Z.; Cao S.; Wei J.; Luo Y.; Xiao Y.; Liu L.; Chen X. Decimal solvent-based high-entropy electrolyte enabling the extended survival temperature of lithium-ion batteries to– 130° C. CCS Chem. 2021, 3 (4), 1245–1255. 10.31635/ccschem.020.202000341.

[ref23] Cho Y. G.; Kim Y. S.; Sung D. G.; Seo M. S.; Song H. K. Nitrile-assistant eutectic electrolytes for cryogenic operation of lithium ion batteries at fast charges and discharges. Energy Environ. Sci. 2014, 7 (5), 1737–1743. 10.1039/C3EE43029D.

[ref24] Baclig A.; Ganapathi D.; Ng V.; Penn E.; Saathoff J.; Chueh W. C. Large Decrease in the Melting Point of Benzoquinones via High-n Eutectic Mixing Predicted by a Regular Solution Model. J. Phys. Chem. B 2023, 127 (27), 6102–6112. 10.1021/acs.jpcb.3c01125. 37384541

[ref25] Mushtaq M.; Butt F. W.; Akram S.; Ashraf R.; Ahmed D. Deep eutectic liquids as tailorable extraction solvents: a review of opportunities and challenges. Crit. Rev. Anal. Chem. 2022, 1–27. 10.1080/10408347.2022.2125284. 36148704

[ref26] Yalkowsky S. H. Carnelley’s rule and the prediction of melting point. J. Pharmaceut. Sci. 2014, 103 (9), 2629–2634. 10.1002/jps.24034. 24899585

[ref27] Lian B.; Yalkowsky S. H. Unified physicochemical property estimation relationships (UPPER). J. Pharmaceut. Sci. 2014, 103 (9), 2710–2723. 10.1002/jps.24033. 24909850

[ref28] Hutchinson J. M. Determination of the glass transition temperature: Methods correlation and structural heterogeneity. Therm. Anal. Calorim. 2009, 98, 579–589. 10.1007/s10973-009-0268-0.

[ref29] Schawe J.; Riesen R.; Widmann J.; Schubnell M.; Jorimann U. UserCom: Information for Users of Mettler-Toledo Thermal Analysis Systems; METTLER-TOLEDO, 2000.

[ref30] Wunderlich B. One hundred years research on supercooling and superheating. Thermochim. Acta 2007, 461 (1–2), 4–13. 10.1016/j.tca.2006.11.015.

[ref31] Cerajewski U.; Träger J.; Henkel S.; Roos A. H.; Brehm M.; Hinderberger D. Nanoscopic structures and molecular interactions leading to a dystectic and two eutectic points in [EMIm] [Cl]/urea mixtures. Phys. Chem. Chem. Phys. 2018, 20 (47), 29591–29600. 10.1039/C8CP04912B. 30328848

[ref32] Malik M.; Ng K. L.; Azimi G. Physicochemical characterization of AlCl_3_- urea ionic liquid analogs: speciation, conductivity, and electrochemical stability. Electrochim. Acta 2020, 354, 136708 10.1016/j.electacta.2020.136708.

[ref33] Böttcher R.; Mai S.; Borisenko N.; Ispas A.; Bund A.; Endres F. A Raman Study on the Speciation of Different Metal Ions in an AlCl_3_–Based Ionic Liquid. J. Electrochem. Soc. 2023, 170 (7), 072503 10.1149/1945-7111/ace383.

[ref34] Abbott A. P.; Qiu F.; Abood H. M.; Ali M. R.; Ryder K. S. Double layer, diluent and anode effects upon the electrodeposition of aluminium from chloroaluminate based ionic liquids. Phys. Chem. Chem. Phys. 2010, 12 (8), 1862–1872. 10.1039/B917351J. 20145853

[ref35] Schoetz T.; Leung O.; de Leon C. P.; Zaleski C.; Efimov I. Aluminium deposition in EMImCl-AlCl_3_ ionic liquid and ionogel for improved aluminium batteries. J. Electrochem. Soc. 2020, 167 (4), 040516 10.1149/1945-7111/ab7573.

[ref36] Bard A. J.; Faulkner L. R.; White H. S. Electrochemical Methods: Fundamentals and Applications; John Wiley & Sons, 2022.

