# Supplementary material for: Ternary Ionic Liquid Analogues as Electrolytes for Ambient and Low-Temperature Rechargeable Aluminum Batteries
Source: ACS Appl Energy Mater. 2024 Jun 20;7(13):5438–46. doi: 10.1021/acsaem.4c00739 (PMC11234329; doi:10.1021/acsaem.4c00739)
Supplement: Supplementary file 1 — ae4c00739_si_001.pdf [file ae4c00739_si_001.pdf]

Supporting Information

Ternary Ionic Liquid Analogues as Electrolytes for Ambient and Low-Temperature Rechargeable Aluminum Batteries

Jonah Wang, Theresa Schoetz, Leo W. Gordon, Elizabeth J. Biddinger*, Robert J. Messinger*

Department of Chemical Engineering, The City College of New York, CUNY, New York, New York 10031, United States

*Email: ebiddinger@ccny.cuny.edu, rmessinger@ccny.cuny.edu

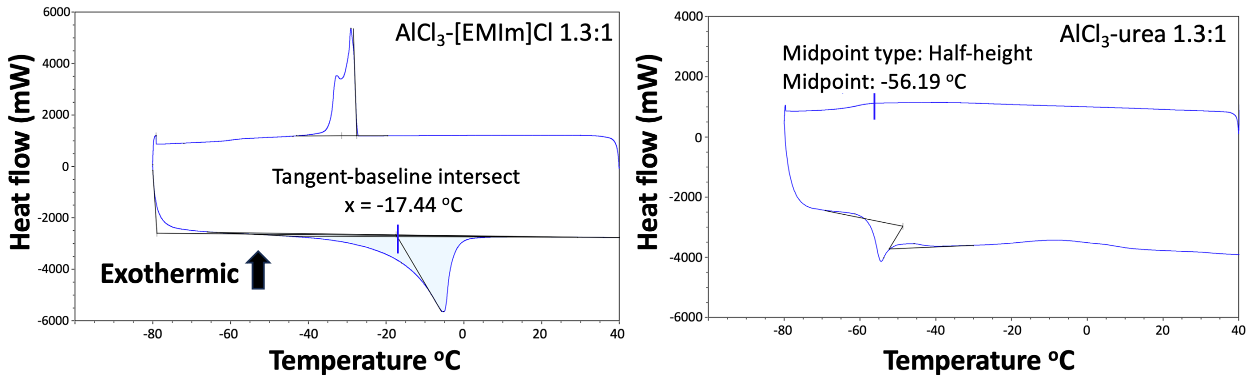

Figure S1. DSC thermograms of a (A) $AlCl_3$-[EMIm]Cl electrolyte with a molar ratio of 1.3:1 and a (B) $AlCl_3$-urea electrolyte with a molar ratio of 1.3:1. The tangent and half-height lines used to calculate the freezing and glass transition temperatures are shown in (A) and (B), respectively.

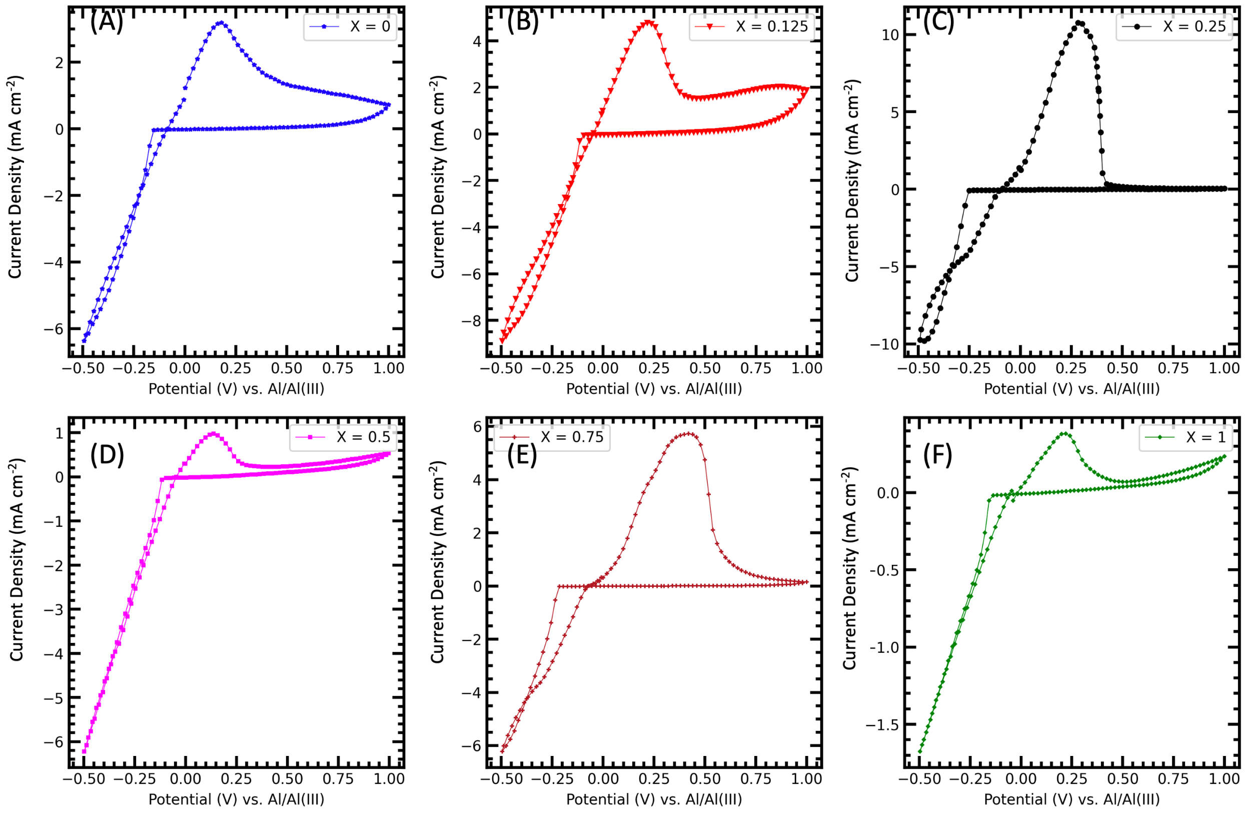

Figure S2. Cyclic voltammetry (CV) performed at 10 mV/s and 25 ºC using $AlCl_3$-urea-[EMIm]Cl electrolytes with molar ratios of 1.3:*X*:(1-*X*), where *X* = (A) 0, (B) 0.125, (C) 0.25, (D) 0.5, (E) 0.75, and (F) 1.0. A three-electrode cell was used with glassy carbon working and counter electrodes and an Al wire quasi-reference electrode. The data is identical to that displayed in Figure 3a (main article), but stacked to enable observation of individual features. Note that the *y*-axis differs between the plots.

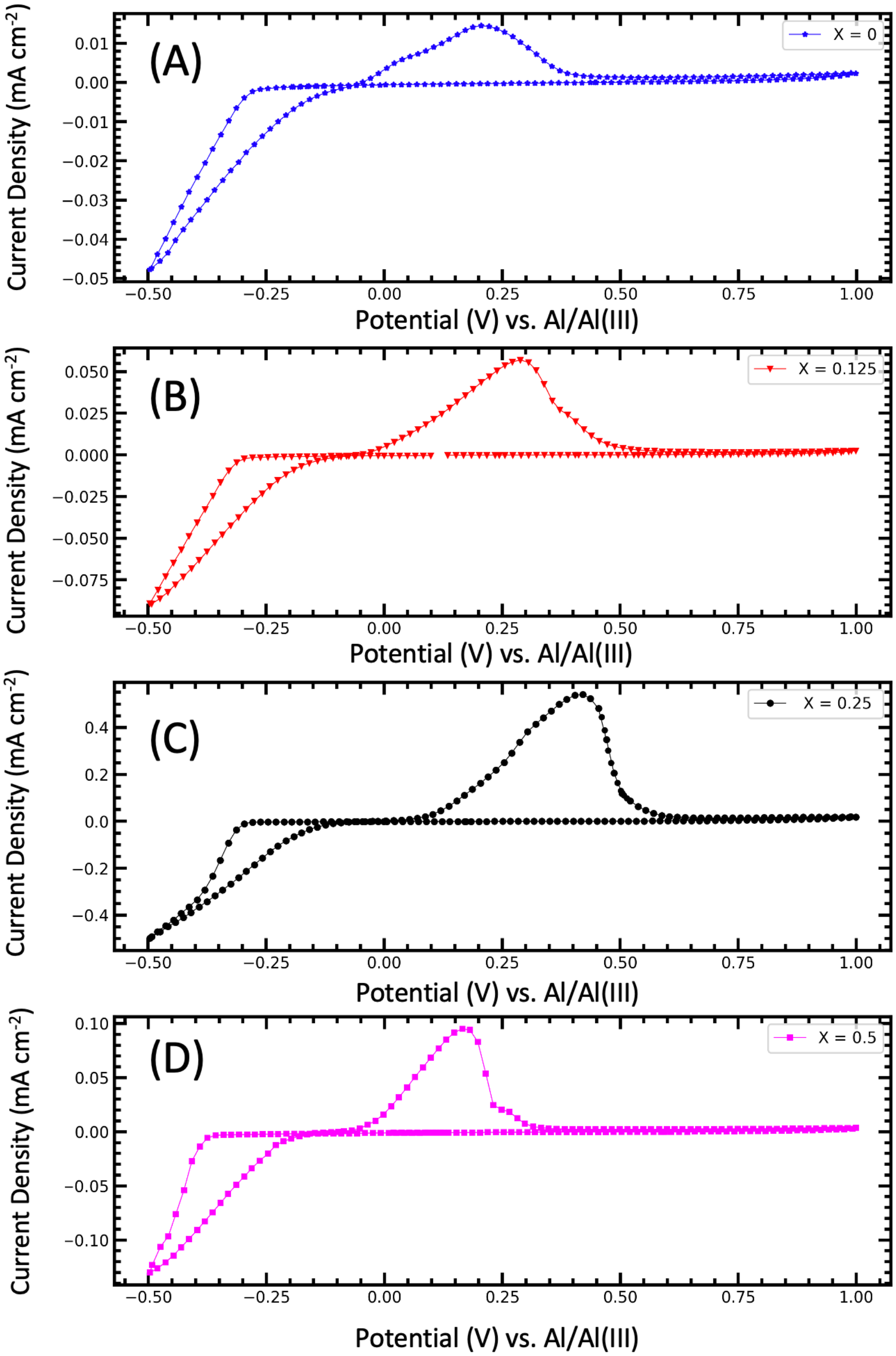

Figure S3. Cyclic voltammetry performed at 10 mV/s and -40 °C using $AlCl_3$-urea-[EMIm]Cl electrolytes with molar ratios of 1.3:X:(1-X), where X = (A) 0, (B) 0.125, (C) 0.25, and (D) 0.5. A three-electrode cell was used with glassy carbon working and counter electrodes and an Al wire quasi-reference electrode. The data is identical to that displayed in Figure 3b (main article), but stacked to enable observation of individual features. Note that the y-axis differs between the plots.

Table S1. Comparison of plating and stripping charge for cyclic voltammograms conducted at 10 mV/s from -0.5 V to 1 V at 25 °C. The $AlCl_3$-urea-[EMIm]Cl electrolyte with a molar ratio of 1.3:0.25:0.75 ($X = 0.25$) is shown to result in the greatest current density. The $AlCl_3$-[EMIm]Cl electrolyte with a molar ratio of 1.3:1 ($X = 0$) has a line shape and coulombic efficiency that match closely to literature results[1] for a $AlCl_3$-[EMIm]Cl electrolyte with a molar ratio of 1.22:1.

Electrolyte	Average Area of plating potential	Average area of stripping potential	Coulombic Efficiency
X = 0	73.16 mAs	42.84 mAs	59%
X = 0.125	114.5 mAs	62.51 mAs	55%
X = 0.25	156.6 mAs	119.58 mAs	77%
X = 0.5	74.70 mAs	14.26 mAs	19%
X = 0.75	68.88 mAs	63.17 mAs	92%
X = 1	19.56 mAs	6.02 mAs	32%

Table S2. Comparison of plating and stripping charge for cyclic voltammograms conducted at 10 mV/s from -0.5 V to 1 V at -40 °C. The $AlCl_3$-urea-[EMIm]Cl electrolyte with a molar ratio of 1.3:0.25:0.75 (X = 0.25) is shown to transfer the greatest quantity of charge upon both plating and stripping. Additional urea content beyond the $AlCl_3$-urea-[EMIm]Cl electrolyte with a molar ratio of 1.3:0.50:0.50 (X = 0.50) resulted in no observable redox activity at this temperature.

Electrolyte	Average Area of plating potential	Average area of stripping potential	Coulombic Efficiency
X = 0	0.47 mAs	0.16 mAs	34%
X = 0.125	0.82 mAs	0.42 mAs	51%
X = 0.25	4.25 mAs	3.78 mAs	89%
X = 0.5	1.17 mAs	0.72 mAs	62%

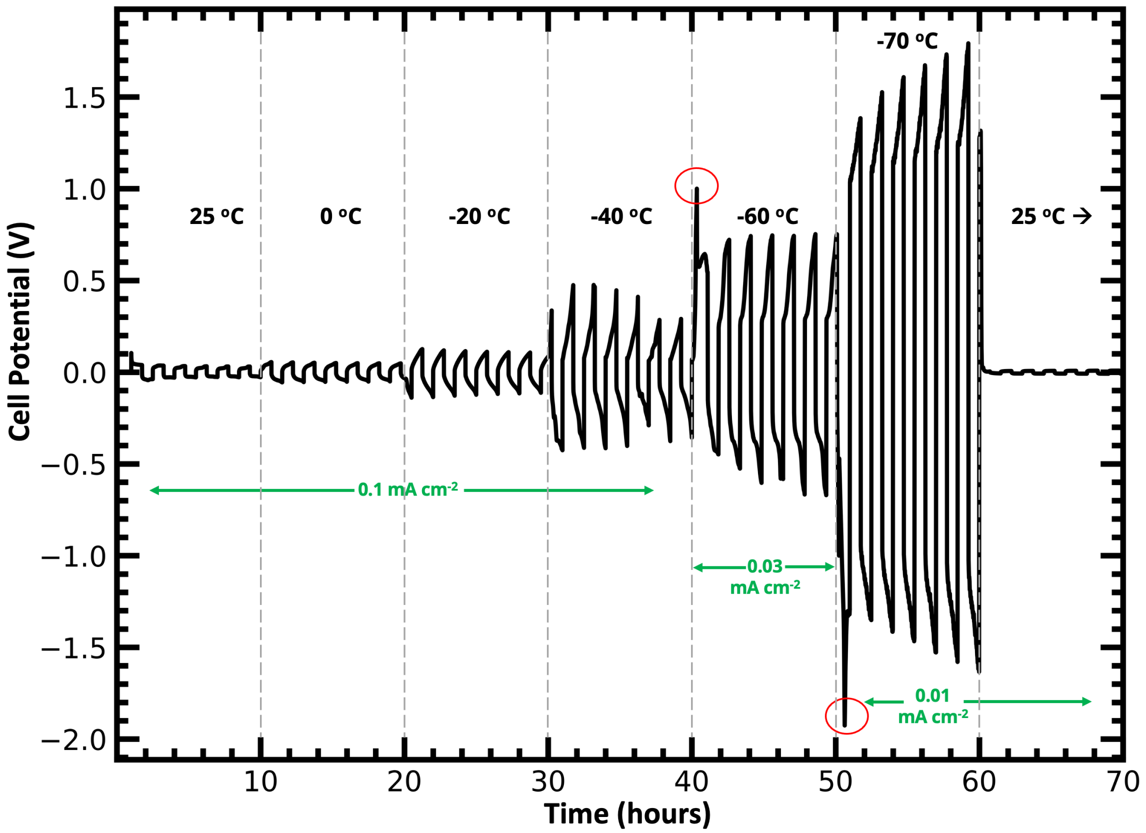

Figure S4. Overpotentials for an Al-Al symmetric cell using a $AlCl_3$-urea-[EMIm]Cl electrolyte with a molar ratio of 1.3:0.25:0.75 ($X = 0.25$) galvanostatically cycled from 25 ºC to -70 ºC, then back to 25 ºC, at current densities of 0.1, 0.03, and 0.01 mA cm^{-2} (as noted in the figure). Voltage limits were set to reduce the current density whenever the cell potential reached a certain threshold, which was 1.0 V for the 0.1 mA cm^{-2} current density and 2 V for the 0.03 mA cm^{-2} and 0.01 mA cm^{-2} current density. The 2 V limit set for the 0.01 mA cm^{-2} current density was not reached, even at -70 ºC.

References

1. T., Leung, O., de Leon, C. P., Zaleski, C., & Efimov, I. Aluminium deposition in EMImCl-$AlCl_3$ ionic liquid and ionogel for improved aluminium batteries. *J. Electrochem. Soc.*, **2020**, *167*(4), 040516.
